# Synthesis and Characterization of Sulfonamide-Containing Naphthalimides as Fluorescent Probes

**DOI:** 10.3390/molecules29122774

**Published:** 2024-06-11

**Authors:** Zhi-Wei Liu, Fan Liu, Chun-Tao Shao, Guo-Ping Yan, Jiang-Yu Wu

**Affiliations:** 1 College of Chemical and Material Engineering, Quzhou University, Quzhou 324000, China; liuhui2015005@sina.com; 2 School of Materials Science and Engineering, Wuhan Institute of Technology, Wuhan 430205, China; 5245934@163.com (F.L.); polymers2012@163.com (C.-T.S.); 15337183953m0@sina.cn (J.-Y.W.)

**Keywords:** fluorescent imaging, fluorescent probe, naphthalimide, sulfonamide, sulfadiazine

## Abstract

A tumor-targeting fluorescent probe has attracted increasing interest in fluorescent imaging for the noninvasive detection of cancers in recent years. Sulfonamide-containing naphthalimide derivatives (SN-2NI, SD-NI) were synthesized by the incorporation of N-butyl-4-ethyldiamino-1,8-naphthalene imide (NI) into sulfonamide (SN) and sulfadiazine (SD) as the tumor-targeting groups, respectively. These derivatives were further characterized by mass spectrometry (MS), nuclear magnetic resonance spectroscopy (^1^H NMR), Fourier transform infrared spectroscopy (FT-IR), ultraviolet–visible spectroscopy (UV), and a fluorescence assay. In vitro properties, including cell cytotoxicity and the cell uptake of tumor cells, were also evaluated. Sulfonamide-containing naphthalimide derivatives possessed low cell cytotoxicity to B16F10 melanoma cells. Moreover, SN-2NI and SD-NI can be taken up highly by B16F10 cells and then achieve good green fluorescent images in B16F10 cells. Therefore, sulfonamide-containing naphthalimide derivatives can be considered to be the potential probes used to target fluorescent imaging in tumors.

## 1. Introduction

In recent years, fluorescent imaging has attracted increasing interest in a potential medical diagnostics modality for noninvasive detection in cancers [1,2,3]. Although fluorescent imaging in thick tissue layers is hampered by low spatial resolution induced by strong light scattering and the absorption of tissues, the recent advances in instrumentation and fluorescent probes have led to the application of fluorescent imaging to be extended from preclinical research to the clinical detection of cancers [4,5,6].

Some compounds, such as indocyanine green (ICG), 5-aminolevulinic acid (5-ALA), and 1,8-Naphthalimide, are evaluated as potential fluorescent probes in FI to improve imaging contrast and sensitivity [7,8,9]. 1,8-Naphthalimide is a fluorescent compound generally used as an organic dye, luminophore, and anticancer agent [10]. However, these widely used fluorescent probes with a low molecular weight are excreted from the body quickly and usually demonstrate no tumor-targeting biodistribution, which possess significant restrictions for the imaging of tumors. So, a tumor-targeting fluorescent probe is an ideal solution to demonstrate a favorable signal-to-background ratio and yield quantitative and spatially and temporally indexed information on both normal tissue and tumors [11].

Some fluorescent probes, such as ICG, IRDye800CW, rhodamine B (RhB), and omocyanine, have been covalently linked to a tumor-targeting molecule with a high affinity to tumors and then produced tumor-targeting fluorescent probes [12,13,14]. Tumor-targeting molecules, including folate, porphyrins, arginine–glycine–aspartic acid (RGD) peptide, and monoclonal antibody targeting epidermal growth factor receptor (EGFR), have been used to make potential tumor-targeting probes for tumor diagnosis and imaging [15,16,17,18,19].

Sulfonamide (SN) and sulfadiazine (SD) derivatives have been investigated as tumor- targeting groups due to their potent and highly selective carbonic anhydrase (hCA IX) inhibitors, which are well-known transmembrane CA isoforms. These CA isoforms are highly expressed in different tumor types and present a rather limited expression in most normal cells [20,21]. Therefore, SN and SD derivatives were reported as the active ingredients in treating cancer and suppressing metastasis. SD derivatives were concentrated into Walker carcinoma or Yoshida sarcoma with 2–3 times as large as the concentration in the liver [21,22,23]. Gadolinium diethylenetriaminepentaacetic acid derivatives containing SD and SN groups were investigated as the potential tumor-targeting contrast agents and possessed high uptake by Hepatoma and Ehrlich ascites carcinoma in mice [24]. Polyaspartamide gadolinium complexes containing SD groups also had specific uptake by Hepatoma and greatly enhanced the contrast of magnetic resonance images of Hepatoma in mice [25].

**Scheme 1:**
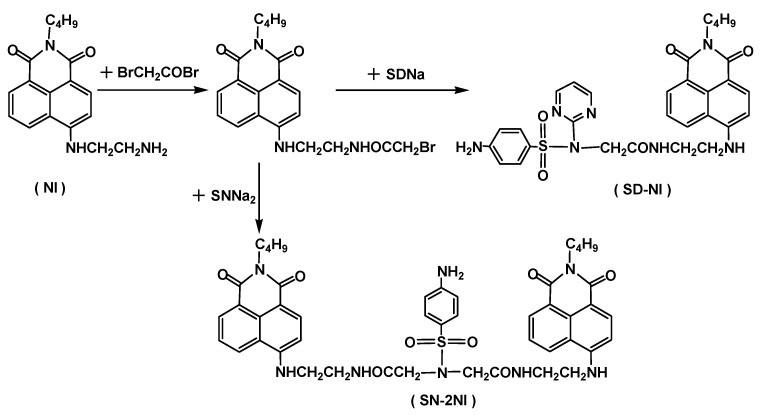
Synthetic route to sulfonamide-containing naphthalimides.

In this work, SD and SN were chosen as the tumor-targeting groups and then incorporated to the N-butyl-4-ethyldiamino-1,8-naphthalimide (NI) [26,27] fluorescent molecule to synthesize tumor-targeting fluorescent probes (Scheme 1). These fluorescent probes were further characterized, and their properties in vitro were also evaluated to find a potential to achieve highly sensitive imaging in tumor cells with high contrast enhancement.

## 2. Results and Discussion

### 2.1. Synthesis

**Figure 1:**
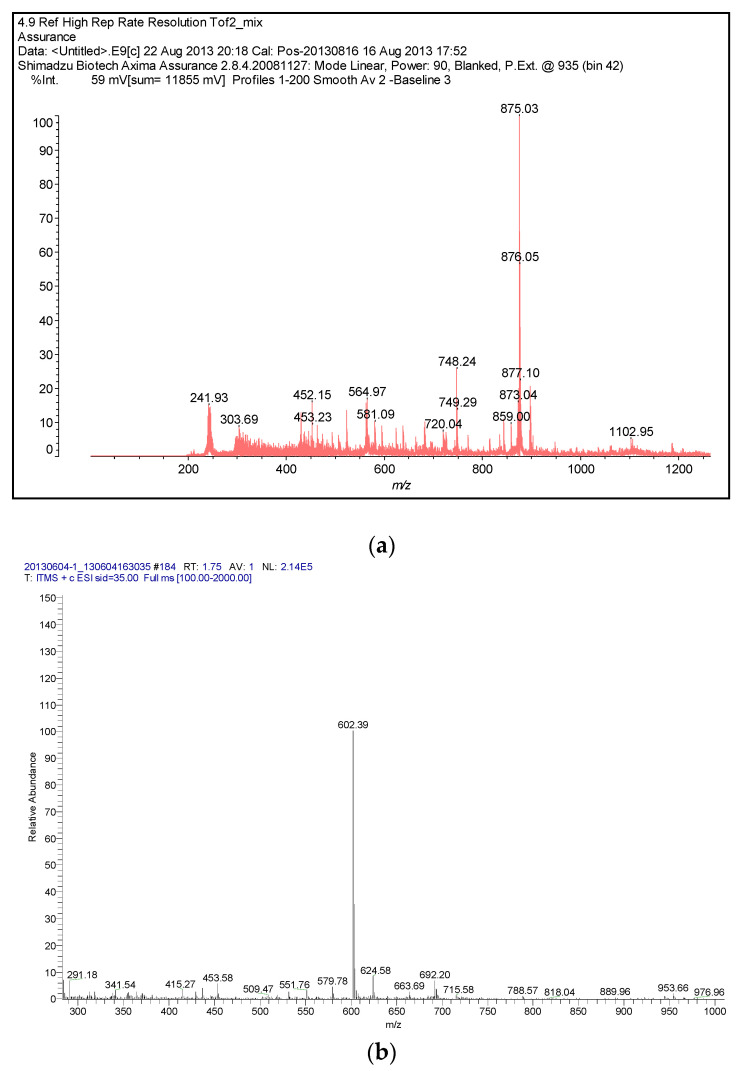
MS spectra of SN-2NI (**a**) and SD-NI (**b**).

**Figure 2:**
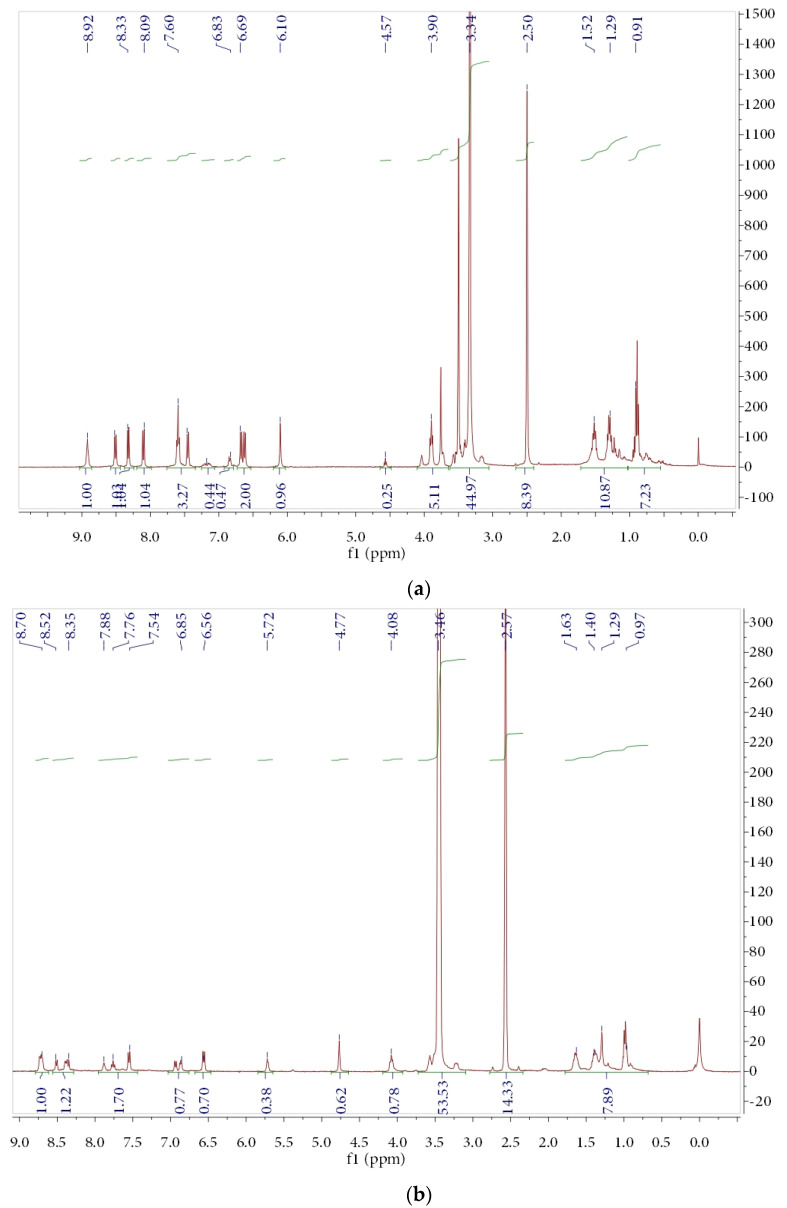
^1^H NMR spectra of SN-2NI (**a**) and SD-NI (**b**).

**Figure 3:**
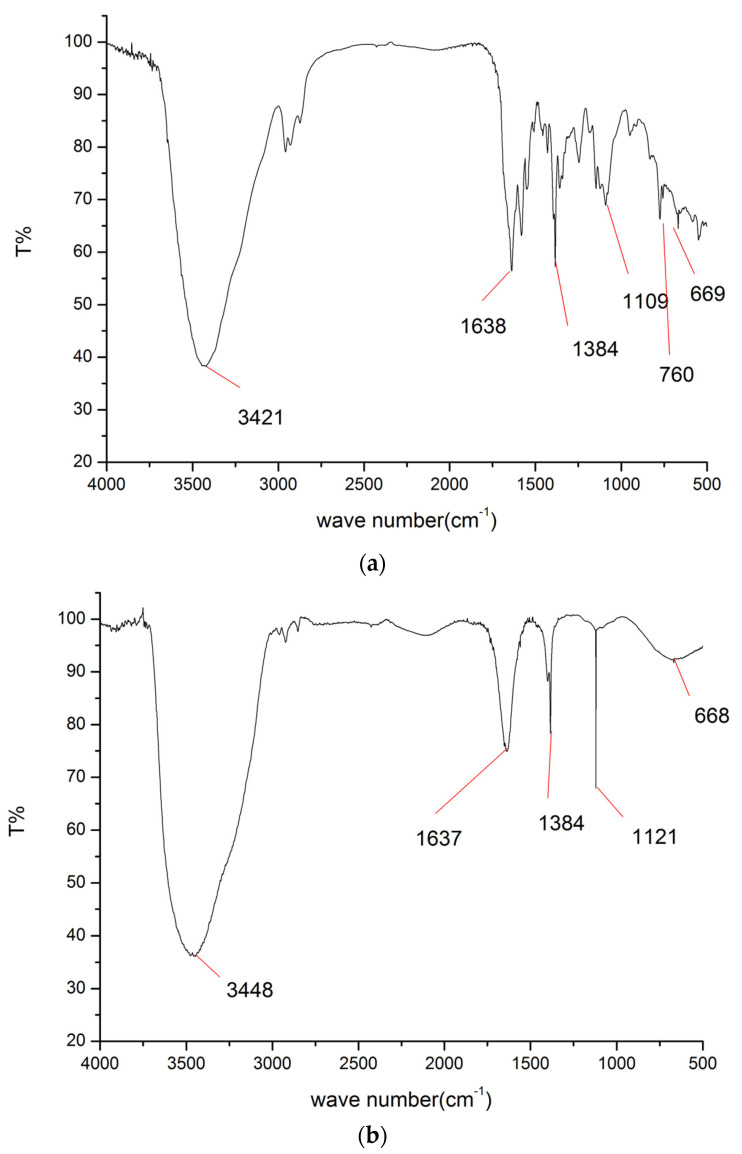
IR spectra of SN-2NI (**a**) and SD-NI (**b**).

**Figure 4:**
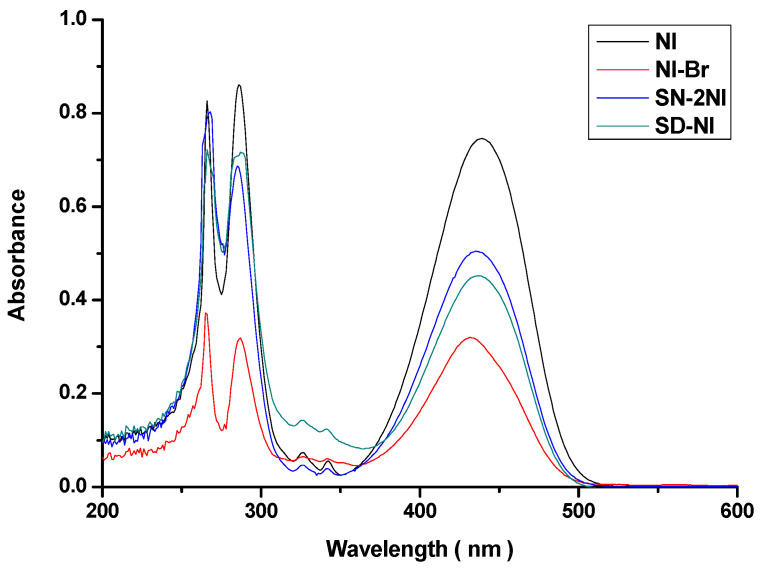
UV spectra of SN-2NI and SD-NI.

**Figure 5:**
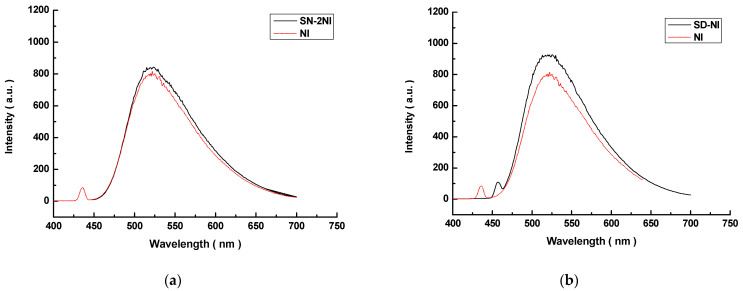
Fluorescent spectra of SN-2NI (**a**) and SD-NI (**b**).

Experimental data, such as FT-IR, ^1^H NMR, UV, MS, and a fluorescent assay, provided evidence for the formation ofsulfonamide-containing naphthalimides (SN-2NI and SD-NI) (Figure 1, Figure 2, Figure 3, Figure 4 and Figure 5), which were synthesized by the incorporation of a NI fluorescent molecule to SN and SD as the tumor-targeting groups (Scheme 1). The MS spectra of SN-2NI displayed the correct [M+H]^+^ peak of 875.03 in accordance with the molecular weight of SN-2NI (MW 874) (Figure 1a). The MS spectra of SD-NI also indicated the correct peak of [M+H]^+^ (602.39), which was consistent with the molecular weight of SD-NI (MW 601) (Figure 1b).

The ^1^H NMR spectra of SN-2NI and SD-NI indicated the typical peaks of naphthalimide ring and methyl and benzyl groups, which appeared at 8.9–8.0 ppm, 1.65–0.8 ppm, and 7.7–7.4 ppm, respectively (Figure 2a,b), indicating that NI was covalently bound to SN and SD, respectively. The IR spectra of SN-2NI and SD-NI showed the characteristic absorption peaks of amide bond (CONH)with 1640–1570 cm^−1^, amino group (NH) at 3450–3420 cm^−1^, and benzyl groups varying from 1150 to 660 cm^−1^, respectively (Figure 3a,b), which indicated that both SN and SD were covalently bound to NI.

SN-2NI and SD-NI displayed similar UV and fluorescent properties as well as NI. The UV spectra of SN-2NI and SD-NI showed the characteristic UV absorption peaks of SD, SN, and NI structures, appearing at 265–267 and 417–435 nm (Figure 4), whist the fluorescent spectra of SN-2NI and SD-NI showed the same characteristic fluorescent property as NI. Meanwhile, SN-2NI and SD-NI had a maximum excitation wavelength at 437 nm and a maximum emission wavelength at 525 nm (Figure 5), respectively. It appears that the typical fluorescent emission wavelength was similar to that of NI. Therefore, SN-2NI and SD-NI are expected to retain the same photophysical properties as their predecessors (NI) to make good fluorescent imaging. Moreover, they should possess a good tumor-targeting property to achieve highly sensitive imaging in tumor cells with high contrast enhancement.

### 2.2. Cell Cytotoxicity

**Figure 6:**
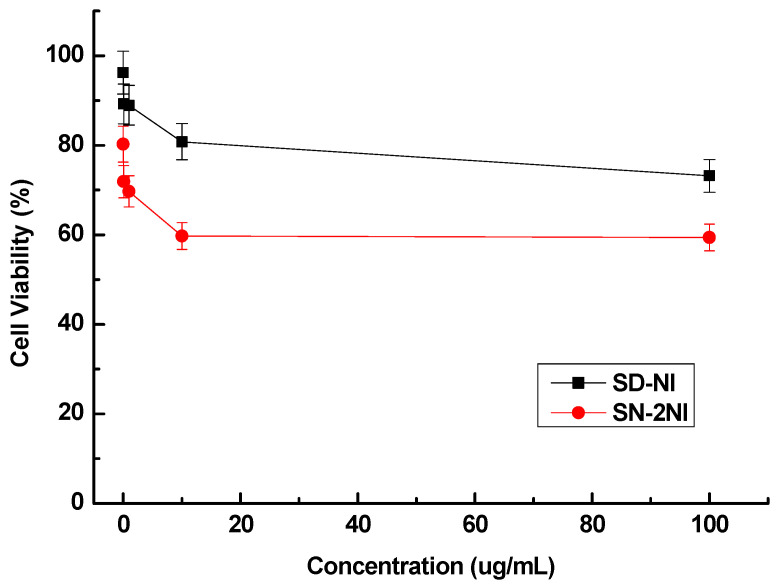
In vitro cytotoxicity assay of SN-2NI and SD-NI to B16F10 cells.

The effect of sulfonamide-containing naphthalimide derivatives (SN-2NI and SD-NI) to B16F10 cell growth and metabolism is shown in Figure 6. At a concentration (1 µg/mL) of SN-2NI and SD-NI in growth medium, the viabilities of B16F10 cells incubated with SN-2NI and SD-NI retained 69.7% and 53.0%, respectively, relative to the control. At the concentration (100 µg/mL) of sulfonamide-containing naphthalimides in the growth medium, the viabilities of B16F10 cells incubated with SN-2NI and SD-NI retained 57.3% and 45.2%, respectively. This illustrated that SN-2NI and SD-NI possessed low cytotoxicity to B16F10 cells and SN-2NI displayed slightly higher cytotoxicity to B16F10 cells than that of SD-NI.

SD and SN are the antibacterial agents and anti-inflammatory drugs used in clinics. Moreover, SD and SN possess good tumor-targeting properties, and then SN-2NI and SD-NI can be selectively taken up by the tumor cells. So, SN-2NI and SD-NI selectively accumulated into B16F10 cells and exhibited obviously high anticancer efficiencies to B16F10 cells when the incubation concentration increased.

### 2.3. Fluorescent Imaging

**Figure 7:**
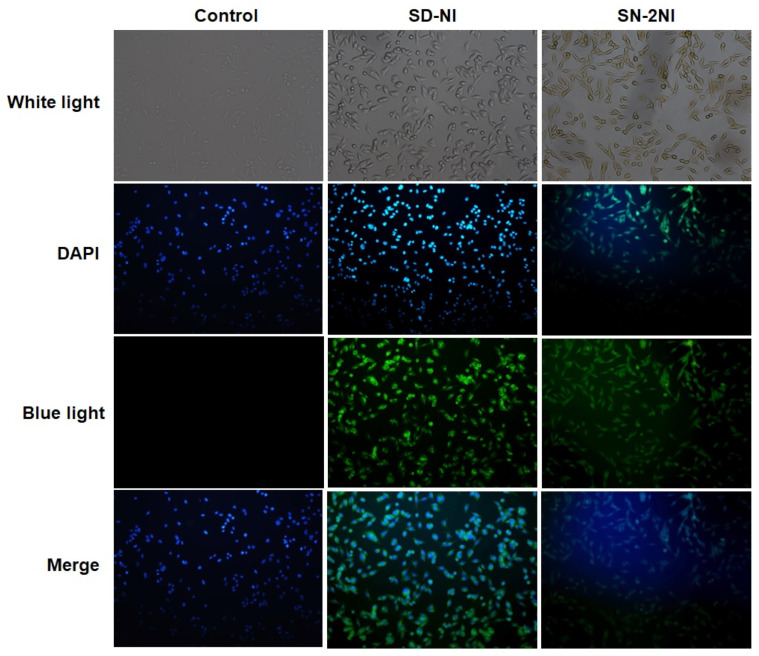
In vitro fluorescent imaging of SN-2NI and SD-NI to B16F10 melanoma cells.

The fluorescent imaging of B16F10 cells cultured with SN-2NI, SD-NI, and pure growth medium was investigated, respectively, when excited by white light (composite excitation wavelength: 400–750 nm, visible light) and blue light (excitation wavelength: 450 nm) (Figure 7).The cells cultured with SN-2NI and SD-NI showed obviously good green fluorescent images when excited by blue light. However, cells cultured with growth medium indicated no fluorescent image in the same condition. Therefore, SN-2NI and SD-NI can be taken highly upby B16F10 cells due to the tumor-targeting property of SN and SD groups.

### 2.4. Cell Uptake and Fluorescent Imaging

**Figure 8:**
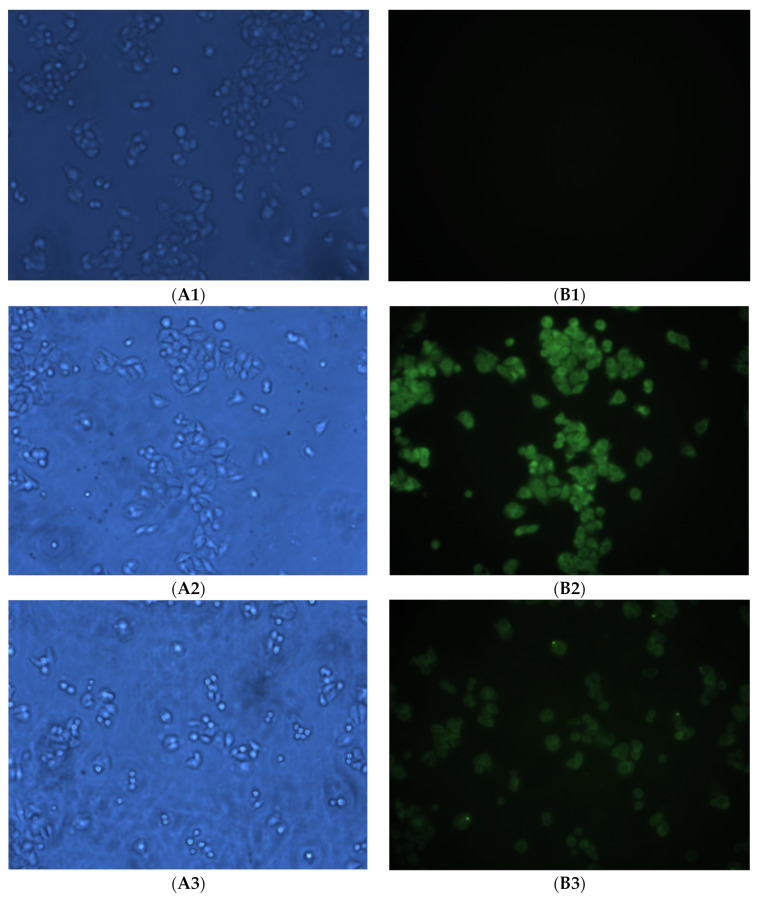
In vitro fluorescent imaging of SN-2NI to B16F10 cells. (**A1** and **B1**: control B16F10 cells excited by white light and blue light, respectively; **A2** and **B2**: B16F10 cells incubated with SN-2NI excited by white light and blue light, respectively; **A3** and **B3**: B16F10 cells were previously incubated by SN (25 μmol/L) for 1 h and subsequently incubated with SN-2NI (0.25 μmol/L) and later excited by white light and blue light, respectively).

**Figure 9:**
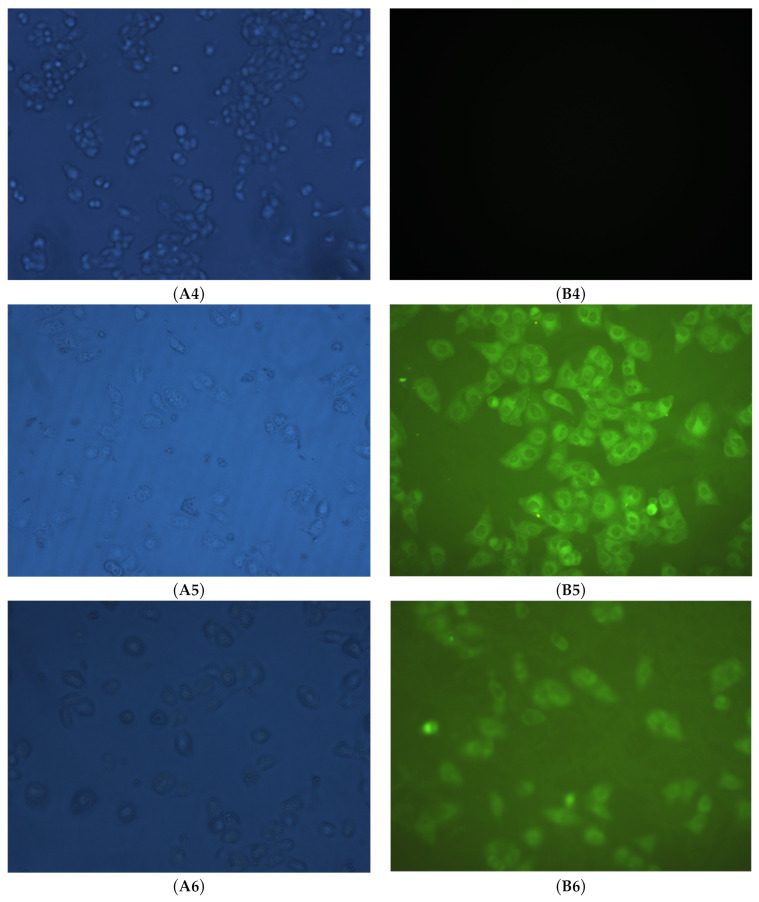
In vitro fluorescent imaging of SD-NI to B16F10 cells.(**A4** and **B4**: control B16F10 cells excited by white light and blue light, respectively; **A5** and **B5**: B16F10 cells incubated with SD-NI excited by white light and blue light, respectively; **A6** and **B6**: B16F10 cells were previously incubated by SD (25 μmol/L) for 1 h and subsequently incubated with SD-NI (0.25 μmol/L) and later excited by white light and blue light, respectively.).

The cell uptake assay and fluorescent imaging of SN-2NI and SD-NI were evaluated to B16F10 cells excited by white light (composite excitation wavelength: 400–750 nm, visible light) and blue light (excitation wavelength: 450 nm) (Figure 8 and Figure 9). Good green fluorescent images can be observed obviously in cells incubated with SN-2NI and SD-NI excited by blue light (Figure 8B2 and Figure 9B5). However, cells incubated with pure growth medium displayed no fluorescent imaging in the same condition (Figure 8B1 and Figure 9B4).

B16F10 cells indicated a significantly lower intensity of green fluorescent images than that of the original cell uptake assaywhen they were incubated previously by SN or SD solution for 1 h, respectively, and by SN-2NI or SD-NI solutions (0.25 μmol/L) later. It is likely that SN or SD covered up earlysome receptor-binding affinity ofSN-2NI and SD-NI in tumor cells. After that, SN-2NI or SD-NI cannot be taken up more or be effectively internalized again by cells. Therefore, SN-2NI and SD-NI possessed good tumor-targeting and characteristic green fluorescent imaging in B16F10 cells via the tumor-targeting property of the SN and SD groups.

## 3. Materials and Methods

### 3.1. Materials

N-Butyl-4-bromoacetyl ethyldiamino-1,8-naphthalimide (NI) [27], sodium sulfadiazine (SDNa), and sodium sulfonamide (SNNa2) [25] were synthesized by the methods cited in the literature. B16F10 mouse melanoma cells were provided by the China Center for Type Culture Collection of Wuhan University, China, and raised according to the method described in the literature [28].

### 3.2. Synthesis of Sulfonamide-Containing Naphthalimide Derivatives (SN-2NI, SD-NI)

A solution of sodium sulfonamide (SNNa, 0.108 g, 0.5 mmol) in N,N-dimethylformamide (DMF, 3 mL) was added slowly to a solution of N-butyl-4-bromoacetyl ethyldiamino-1,8-naphthalene imide (NI, 0.432 g, 1 mmol) in DMF (5 mL) and was stirred at room temperature. Tetrabutyl ammonium hydroxide (40% aqueous solution, 0.3 mL) was also added. The reaction solution continued to be stirred for 24 h at room temperature. Most of the solvent was removed under vacuum, and then the resultant mixture was precipitated with ethanol. The solid residue was filtered and purified by a silica column (an eluent: methanol/dichloromethane:1/3) as to afford brown 4′-bis(N-butyl-4-acetyl ethyldiamino-1,8-naphthaleneimide)-imino-sulfonamide (SN-2NI, 0.27 g, 61.8%) (Scheme 1).^1^H NMR (400 MHz, DMSO-d_6_, δ ppm): 8.92, 8.52, 8.33, 8.09 (d, 4H), 7.61, 7.46, 7.15 (s, 8H), 6.10 (s, 2H), 4.50 (s, 2H), 4.08 (m, 2H), 3.73–3.16 (s, 12H), 1.52, 1.27, 0.89 (m, 18H); MS found [M+H]^+^ 875.03 for C_46_N_8_O_8_H_50_S, calc. 874; IR (KBr, ν_max_, cm^−1^): 3421 (NH), 2958 (C-H), 1638, 1581(CONH), 1557, 1429 (C-H), 1384 (C-N), 1148, 760, 669 (phenyl); UV (H_2_O, λ_max_, nm): 265, 435.

A solution of sodium sulfadiazine (SDNa, 0.544 g, 2 mmol) in acetonitrile (10 mL) was added slowly to a solution of NI (0.864 g, 2 mmol) in acetonitrile (5 mL) and then stirred at room temperature. Tetrabutyl ammonium hydroxide (40% aqueous solution, 0.5 mL) was also added. The reaction solution continued stirring for 24 h at room temperature. Most of the solvent was removed under vacuum and precipitated with ethanol. The solid residue was filtered and purified by a silica column using chloroform as an eluent to make brown 5-(4′-bis(N-butyl-4-acetyl ethyldiamino-1,8-naphthalene imide)-imino-sulfadiazine (SD-NI, 0.8 g, 66.6%) (Scheme 1).^1^H NMR (400 MHz, DMSO-d_6_, δ ppm): 8.71–8.37(d, 4H), 7.88, 7.76, 7.56 (s, 3H), 6.96, 6.87, 6.57 (s, 3H), 5.71 (s, 1H), 4.77 (s, 1H), 4.50 (s, 1H), 4.08 (m, 1H), 1.65, 1.29, 0.98 (m, 9H); MS found [M+H]^+^ 602.39 for C_30_N_7_O_5_H_31_S, calc. 601; IR (KBr, ν_max_, cm^−1^): 3448 (NH), 2923 (C-H), 1637 (CONH), 1572 (C-H), 1384 (C-N), 1121, 668 (phenyl); UV (H_2_O, λ_max_, nm): 267, 417.

### 3.3. Fluorescent Spectroscopy

Sulfonamide-containing naphthalimide derivatives (SN-2NI, SD-NI) were first dissolved in a minimum volume of CHCl_3_ and then diluted with methanol to the desired concentration. Fluorescent spectra were recorded on a Varian Cary Eclipse fluorescence spectrophotometer (Varian, Palo Alto, CA, USA).

### 3.4. In Vitro Cell Cytotoxicity Assay

The RPMI-1640 media (10% fetal bovine serum (Gibco.Co., Billings, MT, USA), 100units/mL penicillium and 100 µg/mL streptomycin)were used to culture B16F10 cells (2 × 10^5^/mL)in vitro using a cell cytotoxicity assay. The cells (2 × 10^4^) were plated in each well of 96-well plates and incubated for 24 h in an incubator (37 °C, 5% CO_2_). The growth medium was replaced with SN-2NI or SD-NI solutions (100 µL) in growth medium. After a 48 h incubation, cells were added to MTT (thiazolyl blue (3-[4,5-dimethylthiazol-2-yl]-2,5-diphenyltetrazolium bromide, 5.0 mg/mL, 20 µL) solution in phosphate-buffered saline solution (PBS) and continued to be incubated for 4 h. Dimethyl sulfoxide (DMSO, 100 µL) was then replaced and shaken for 30 min at room temperature. The absorbance (optical density: OD492) was evaluated at 492 nm by a DG-3022A ELISA-Reader (Hercules, CA, USA), and cell viability was calculated.

### 3.5. Fluorescent Imaging Assay

The DMEM media (100 µg/mL streptomycin, 10% fetal bovine serum and 100 units/mL penicillium) were used to culture B16F10 cells (1 × 10^5^/mL) in vitro using a fluorescent imaging assay. The cells were plated in each well of 6-well plates and incubated for 48 h in an incubator (37 °C, 5% CO_2_). The growth medium was replaced with SN-2NI or SD-NI solutions (1 mL, 100 µg/mL) in a growth medium or pure growth medium, respectively. After 2 h of incubation, the growth medium was removed and the cells were rinsed with PBS three times. And then the cells were fixed with paraformaldehyde (0.5 mL, 4%) for 10 min and stained with 4′,6-diamidino-2-phenylindole (DAPI, 5 μg/mL) for another 10 min. The cell morphology and density were observed in the Leica confocal laser scanning microscope (CLSM) (TCS SP8, Heidelberg, Germany).

### 3.6. Cell Uptake Assay

B16F10 melanoma cells (1 × 10^5^/mL) were seeded in 24-wellplates in the DMEM media (10% fetal bovine serum (Gibco. Co., Chagrin Falls, OH, USA), 100 units/mL penicillium and 100 µg/mL streptomycin), respectively. These cells were incubated for 24 h in an incubator (37 °C, 5% CO_2_), and the growth medium was then replaced with SN-2NI, SD-NI solutions in growth medium or pure growth medium (200 µL), respectively. Cells were incubated for 2 h, washed using PBS three times, and then fixed with paraformaldehyde (0.5 mL, 4%) for 10 min. The cell morphology and density were observed in an IX-70 inverted fluorescence microscope (Olympus Co., Ltd., Tokyo, Japan).

An inhibited cell uptake assay was carried out as the above cell uptake assay. Before the addition of SN-2NI, SD-NI solutions, or pure growth medium (200 µL), cells were previously incubated with SN or SD solutions in growth medium (25 μmol/L, 200 µL), respectively, for 1 h.

## 4. Conclusions

Sulfonamide-containing naphthalimides SN-2NI and SD-NI were synthesized and evaluated as potential fluorescent imaging probes. SN-2NI and SD-NI displayed similar UV and fluorescent properties, as well as NI. Moreover, these sulfonamide-containing naphthalimides possessed low cell cytotoxicity to B16F10 cells. SN-2NI and SD-NI can be taken up highly by B16F10 cells and then achieve good green fluorescent images in B16F10 cells. Therefore, sulfonamide-containing naphthalimides can be considered to be potential probes for fluorescent imaging in tumors.

## Data Availability

Data are contained within the article.
